# A kinetic model of TBP auto-regulation exhibits bistability

**DOI:** 10.1186/1745-6150-5-50

**Published:** 2010-08-05

**Authors:** Sucheta A Gokhale, Reema Roshan, Vivek Khetan, Beena Pillai, Chetan J Gadgil

**Affiliations:** 1 Chemical Engineering and Process Development Division, National Chemical Laboratory, CSIR, Pune 411008, India; 2 Institute of Genomics and Integrative Biology, CSIR, Mall Road, Delhi, 110007, India; 3 Chemical Engineering Department, Indian Institute of Technology Kharagpur, Kharagpur, 721302, India

## Abstract

**Background:**

TATA Binding Protein (TBP) is required for transcription initiation by all three eukaryotic RNA polymerases. It participates in transcriptional initiation at the majority of eukaryotic gene promoters, either by direct association to the TATA box upstream of the transcription start site or by indirectly localizing to the promoter through other proteins. TBP exists in solution in a dimeric form but binds to DNA as a monomer. Here, we present the first mathematical model for auto-catalytic TBP expression and use it to study the role of dimerization in maintaining the steady state TBP level.

**Results:**

We show that the autogenous regulation of TBP results in a system that is capable of exhibiting three steady states: an unstable low TBP state, one stable state corresponding to a physiological TBP concentration, and another stable steady state corresponding to unviable cells where no TBP is expressed. Our model predicts that a basal level of TBP is required to establish the transcription of the TBP gene, and hence for cell viability. It also predicts that, for the condition corresponding to a typical mammalian cell, the high-TBP state and cell viability is sensitive to variation in DNA binding strength. We use the model to explore the effect of the dimer in buffering the response to changes in TBP levels, and show that for some physiological conditions the dimer is not important in buffering against perturbations.

**Conclusions:**

Results on the necessity of a minimum basal TBP level support the in vivo observations that TBP is maternally inherited, providing the small amount of TBP required to establish its ubiquitous expression. The model shows that the system is sensitive to variations in parameters indicating that it is vulnerable to mutations in TBP. A reduction in TBP-DNA binding constant can lead the system to a regime where the unviable state is the only steady state. Contrary to the current hypotheses, we show that under some physiological conditions the dimer is not very important in restoring the system to steady state. This model demonstrates the use of mathematical modelling to investigate system behaviour and generate hypotheses governing the dynamics of such nonlinear biological systems.

**Reviewers:**

This article was reviewed by Tomasz Lipniacki, James Faeder and Anna Marciniak-Czochra.

## Background

Genetic, metabolic and signalling regulatory networks show different types of regulatory modes such as positive and negative feedback that lead to non-intuitive phenotypic properties such as multistability, oscillations and hysteresis [1-3]. Auto-catalysis, where a molecule enhances its own production or activity, is one of the modes of autogenous regulation [4]. This type of regulation can lead to multistability due to the nonlinear nature of the feedback. In many cases this is achieved through binding of the protein product to a regulatory site upstream of its gene. In some cases [5,6], the protein forms a dimer, and binds to the DNA in dimeric form. In other cases [7], the protein does not dimerize and binds to DNA as a monomer. Both these motifs have been studied experimentally and through mathematical models [8,9]. The interaction of the transcription factor TATA Binding Protein (TBP) with eukaryotic promoters differs from these forms.

TBP is a ubiquitously expressed general transcription factor which binds to a promoter element called the TATA box. The TATA box is represented by the consensus sequence TATAAT that occurs at about 30 to 40 base pairs (bp) upstream of the transcription start site. TBP binding to the TATA box nucleates the assembly of the other transcription factors and the RNA polymerase. There is another class of promoters (TATA-less promoters) that lack the canonical TATA box. The promoter for the TBP gene is thought to be a TATA-less promoter [10,11]. RNA pol II requires TFIID for transcription from such promoters. TBP is known to be integral component of TFIID. TBP interacts with such promoters indirectly through tethering factors [12]. Promoters of genes transcribed by RNA pol I and pol III are generally TATA-less. In case of transcription by RNA pol I TBP forms complex with SL1 protein while in transcription by RNA pol III TBP forms a part of TFIIB complex [13]. Studies have shown that inactivation or depletion of TBP leads to rapid decrease in transcription by all three polymerases [14,15]. Therefore, irrespective of the presence of TATA box, TBP is required for eukaryotic transcription initiation by all the three RNA polymerases [16]. X-ray crystallographic structure shows that the molecule is saddle shaped. Its concave surface interacts with the minor groove of DNA and its convex surface interacts with transcription factors.

TBP dimerizes in solution but the dimer is incapable of binding to DNA. It is the monomer that binds to DNA and this binding is an important step in transcriptional activation at the majority of eukaryotic promoters. Although TBP-DNA binding has been modelled [17], the effect of auto-catalysis and the effect of dimer fraction has not been studied under physiological conditions through experiments or modelling. We present the first mathematical model of TBP that includes auto-catalytic formation and negative regulation of its own activity by dimerization. We show that TBP levels are sensitive to parametric changes under many of the physiological conditions and a certain minimum amount of TBP is required for cell viability.

**Figure 1:**
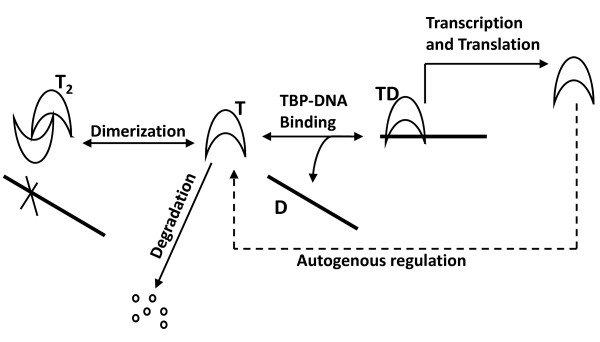
**A schematic representation of steps involved in auto-regulation of TBP**. TBP exists in solution as dimer, but binds to DNA as a monomer. The filled lines represent DNA and the arc shapes represent TBP molecules.

During dimerization of TBP the DNA binding concave surface is sequestered, hence dimerization of TBP hinders its binding to DNA [16,18]. TBP dimers are quite stable and have an equilibrium constant in the nanomolar range (k_D _= 5*10^-10 ^M) [19]. This slow dissociation of dimers represents a rate limiting step in DNA binding. TBP dimerization competes with DNA binding activity. This suggests that dimerization is a mechanism for negatively auto regulating DNA binding activity [17] and thus preventing unregulated gene expression [20]. Under physiological conditions, TBP exists as a dimer when not bound to DNA [19]. In vitro experiments by Pugh and co-workers showed that TBP dimer concentration decreased with increasing oligonucleotide concentration, which indicated that there is high affinity of TBP for DNA [19]. We use the model developed here (Figure 1, Table 1, Table 2) to computationally test the effect of the dimer on maintaining TBP levels at physiological conditions.

**Table 1:** Reactions used in model

Reaction	Rate equation
T + T → T_2_	*k*1 × [*T*]^2^

T_2 _→ T + T	*k*2 × [*T*_2_]

T + D → TD	*k*3 × [*T*] ×[*D*]

TD → T + D	*k*4 × [*TD*]

Φ → T	$k0+\frac{k5\times {\left[TD\right]}^{k6}}{k7^{k6}+{\left[TD\right]}^{k6}}$

T → Φ	*k*8 × [*T*]

**Table 2:** Parameter values used for simulation

Parameter value	Reference
k1 = 1*10^5 ^M^-1^s^-1^	[17]

k2 = 1*10^-3 ^s^-1^	[17]

k3 = 2*10^5 ^M^-1^s^-1^	[17,19,36]

k4 = 4*10^-4 ^s^-1^	[36]

k5 = 5*10^-13 ^Ms^-1^	From [37] and k8

k7 = 1.25*10^-8^M	Half of D_0_

k8 = 7.4*10^-5 ^s^-1^	[20]

k6 = 2	Assumed

k0 = 5*10^-15 ^Ms^-1^	Assumed (0.01*k5)

[D_0_] = 2.5*10^-8 ^M	[34]

## Results

### Minimum amount of initial TBP is required for cell viability

**Figure 2:**
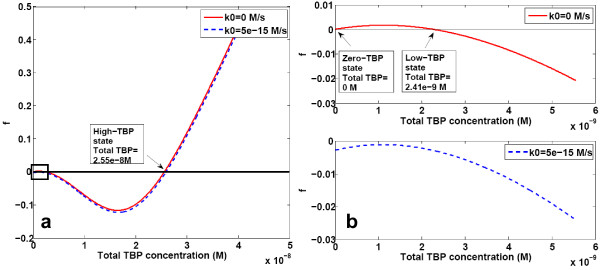
**Graphical solution for TBP steady state**. **(a) **The function of TBP concentration described by equations 6 and 7 was plotted against total TBP concentration. **(b) **Magnified view of the region enclosed in the square in 2a. Parameter values are as given in Table 2.

The TBP system, as several other positive feedback systems, showed the presence of multiple steady states. The equation for the steady state TBP concentration (equation 6) was solved graphically by plotting values of function f against the equivalent total TBP concentration (Figure 2) and identifying the points at which the graph intersects the x-axis (i.e. f = 0). In the presence of a basal transcription rate (k0 > 0), only one steady state was seen. Considering the requirement of TBP for all the three polymerases, it was reasonable to assume that no transcription occurs in the absence of TBP, i.e. TBP production rate was equal to zero in the absence of TBP, or k0 = 0. In the absence of basal expression, the graph showed existence of three steady states in the system. Those correspond to total TBP concentration of 0 M, 2.41*10^-9^M and 2.55*10^-8^M, i.e. a zero-state, a low-TBP state, and a high-TBP state. For different parameter values, the numerical values of the nonzero states changed, but henceforth the highest-concentration state is referred to as the high-TBP state and the state corresponding to the non-zero TBP concentration less than the high-TBP state is called the low-TBP state. The sole stable steady state seen in the presence of a basal transcription was almost identical to the high-TBP steady state observed in the absence of the basal transcription.

**Figure 3:**
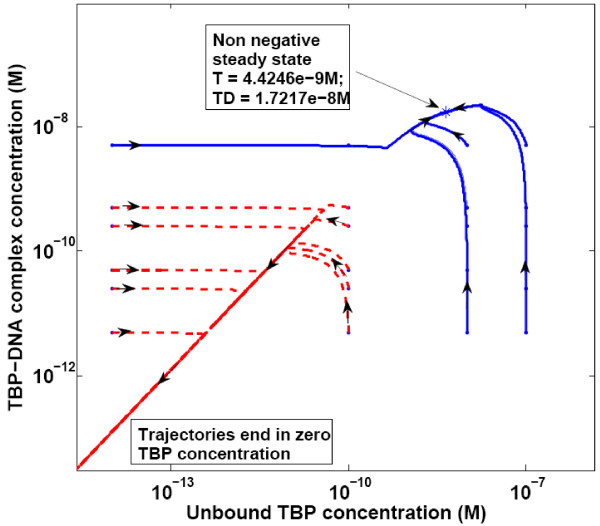
**Phase plane plot**. Bistable nature of the system illustrated by phase plane plot. At different starting concentrations, two stable states were seen. Parameter values are as given in Table 2.

From a linear stability analysis for parameter values corresponding to a typical mammalian cell, the low-TBP state was found to be unstable, whereas the zero-state and the high-TBP state were stable steady states. The trajectories leading to these stable steady states are shown in a phase plane plot of TBP-DNA and unbound TBP concentration (Figure 3). The phase plane plot showed that starting from different initial conditions; the system reached one of the two stable steady states, confirming the bistable nature of the system. It was seen that below a certain total TBP level, all trajectories led to the zero-state, suggesting that a minimum TBP concentration was required for the system to reach the physiological high-TBP steady state and hence for cell viability.

The time course trajectories of unbound TBP and TBP-DNA complex concentrations were found to meet into a line. This nature of the phase plane plot was due to the fast TBP-DNA binding and slow TBP formation and degradation reactions. The line represented the (pseudo)-equilibrium concentrations of unbound TBP and TBP-DNA complex. The ratio of concentrations ([T]*[D])/[TD]) for values corresponding to the straight line region of trajectories was calculated and found to be equal to the equilibrium dissociation constant for TBP-DNA binding.

### Sensitivity to parameter variation

**Figure 4:**
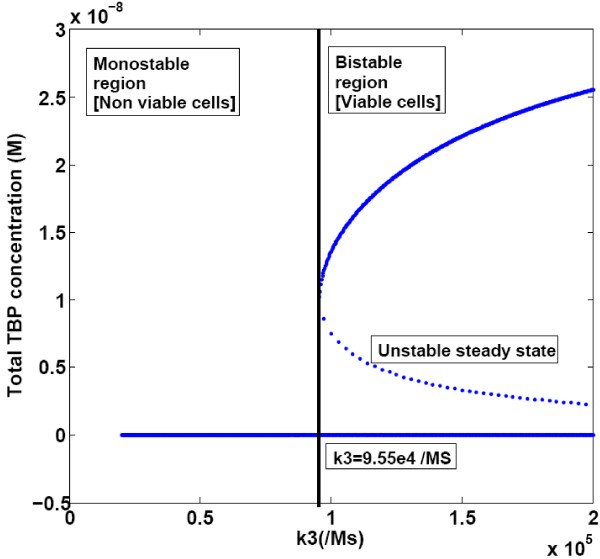
**Bifurcation diagram of TBP- DNA binding rate constant (k3)**. Reaction rate constant for TBP-DNA binding reaction (k3) was varied in the simulations, Other parameters were kept constant at values reported in Table 2.

The steady state concentration of TBP was calculated at various values of the DNA binding and synthesis rate constants to determine how the steady state values change as a function of parameter value. Sensitivity was tested qualitatively and quantitatively. Change in number of fixed points and their stability was analysed to qualitatively test the sensitivity to parameter variation. Quantitatively the relative change in the value of high-TBP state corresponding to a change in parameter value was calculated. The plot of total TBP steady state concentration vs. the TBP- DNA association reaction rate constant (k3) (Figure 4) showed that for values of k3 upto 9.556*10^4 ^M^-1^s^-1 ^there exists only one stable steady state corresponding to 0 M total TBP concentration (the zero-state). For k3 values above this bifurcation value (k3_c), there were two stable steady states, the zero-state and the high-TBP state, and an unstable steady state (the low-TBP state). Thus for values of k3 above 9.556*10^4 ^M^-1^s^-1^, the evolution of the system can result in either the zero or the high TBP concentration state depending on the initial conditions, as seen in Figure 4.

From Figure 4 it was observed that at bifurcation value the high TBP steady state and the low TBP steady state were equal. From the analytical expression for steady states of TBP, the expression for the critical value of k3 (k3_c) was found out.

$$K3\_c=\frac{2\left(K4K5K7^{2}+\sqrt{K4^{2}K5^{2}K7^{2}+K4^{2}K5^{2}K7^{4}}\right)}{K5^{2}}$$

The parameters are dimensionless (Additional file 1, Table S1A).

For parameter values corresponding to those in Table 2, the value for k3_c was calculated to be 9.58*10^4^M^-1^s^-1^, which was comparable to the numerical results.

**Figure 5:**
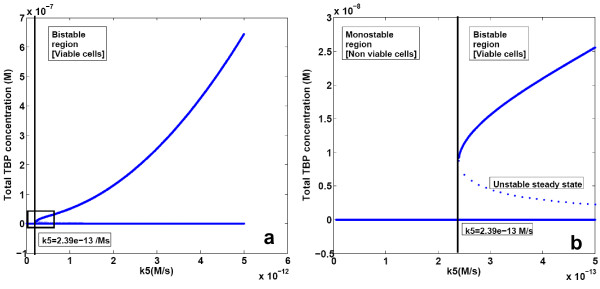
**Bifurcation diagram of TBP synthesis reaction rate constant (k5)**. **(a) **Reaction rate constant for TBP synthesis reaction (k5) was varied in the simulations. Other parameters were kept constant at values reported in Table 2. **(b) **Magnified view of the region enclosed in the square in 5a.

A similar effect was observed on varying the TBP synthesis reaction rate constant (k5). For k5 above a critical value (k5_c) of 2.394*10^-13 ^M/s there were two stable states corresponding to zero TBP and a high TBP concentration and one unstable steady state of low TBP concentration (Figure 5). For k5 below this value only one stable steady state corresponding to zero TBP concentration was observed. The expression for k5_c is given as,

$$K5\_c=2\left(KK7^{2}+\sqrt{K^{2}K7^{2}+K^{2}K7^{4}}\right)$$

The value of k5_c was found to be 2.39*10^-13^M/s, same as that obtained from numerical simulations.

**Figure 6:**
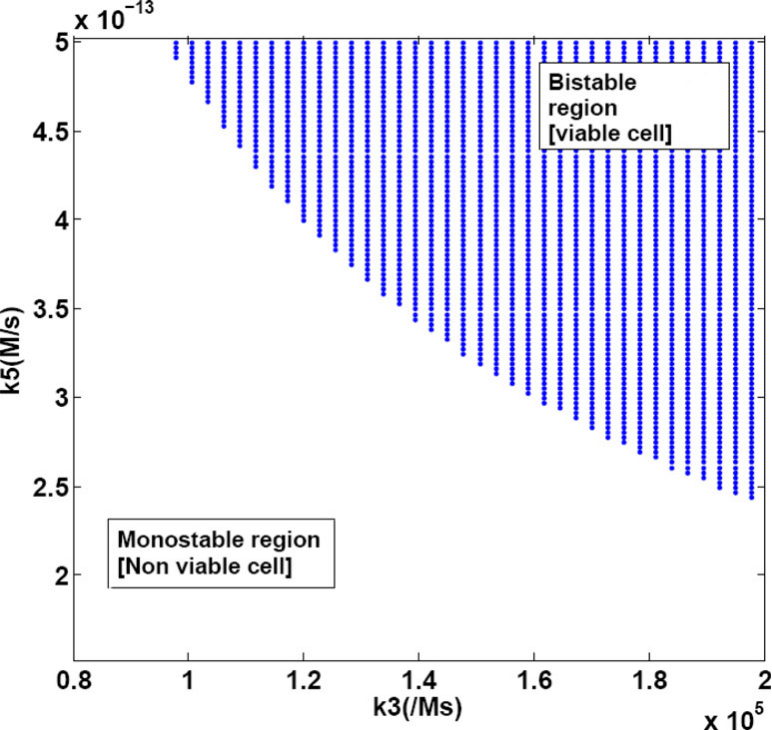
**The monostable and bistable region for different parameter values of k3 and k5**. Two parameters k3 and k5 were varied simultaneously in the simulations. Other parameters were kept constant at values reported in Table 2.

Simultaneously varying both TBP-DNA binding rate constant (k3) and rate constant of TBP synthesis reaction (k5) showed two regions, a monostable region of zero-TBP concentration and a bistable region of high-TBP and zero-TBP concentration (Figure 6).

**Figure 7:**
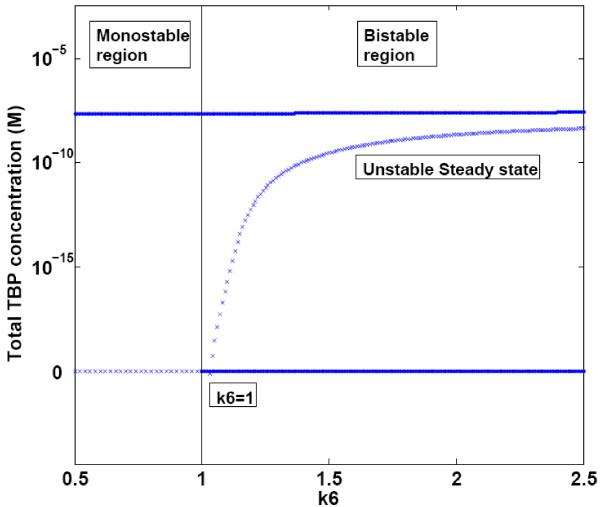
**Bifurcation diagram for Hill cooperativity constant (k6)**. Hill cooperativity coefficient (k6) was varied in the simulations. Other parameters were kept constant at values reported in Table 2.

Changing the Hill cooperativity coefficient (k6) also affected the system properties. For values of k6 above 1 three steady states were observed, with two stable and an unstable steady state, as seen in Figure 7. For the values of k6 below 1 there were two steady states corresponding to zero TBP and high TBP concentrations respectively. In this case the steady state corresponding to high TBP was found to be the stable steady state.

To identify the conditions for multistability, analytical expressions for steady state level of TBP were obtained. The expressions for steady state levels of TBP were analysed for different k0 (basal synthesis rate) and k6 values, for existence of multiple steady states. These conditions are summarized in Table 3. The details are given in Additional file 1. The critical value of k0 (k0_c) was found out such that for k0 < k0_c, there were three steady states. The value of k0_c was found to be 0.008*k5 (4*10^-15^Ms^-1^).

**Table 3:** Conditions for existence of multiple steady states

	K0 = 0	K0 ≠ 0
**k6 = 2**	3 real positive steady states (K5 > 2K7^2^K4/K3 andK7 < 1)	1 real positive steady state (K0 > K0_c) 3 real positive steady states (K0 < K0_c)

**k6 = 1**	2 real positive steady states (K5 > K7K4/K3)	1 real positive steady state (K0 << K and K5 > K7K4/K3)

The parameters are dimensionless.

In the simulations, it was seen that the high-TBP state corresponding to the set of physiological parameters was sensitive to variation in TBP-DNA binding strength (k3) and to TBP synthesis rate (k5), while it was less sensitive to variation in k6. For instance, 50% decrease in k3 value reduced the high-TBP state concentration by ~47%, similarly for 50% reduction in k5 there was ~55% decrease in TBP concentration. On the other hand, decreasing the value of k6 by 50% reduced the high-TBP state concentration by ~9%. For almost all the 23 conditions, a large variation in sensitivity to k3 was observed. The range for reduction in TBP concentration was 0.08% to 52%. The conditions for which the high-TBP state was not sensitive to k3 were physiological extreme conditions except for case 9 which indicated typical yeast cell physiological condition. In case of sensitivity to k6, the range for concentration change in high-TBP state was 0.08% to 28% except for case 21, which unexpectedly showed 21 times increase. Apart from case 18 (28% reduction) and case 22 (22% reduction), variation in TBP concentration for other cases was up to 10%. Cases 21 and 22 were physiological extreme conditions while condition 18 was near to mammalian physiological condition. Details of parameter sensitivity are given in Additional file 1, Table S3A.

### Effect of presence of TBP dimer

**Figure 8:**
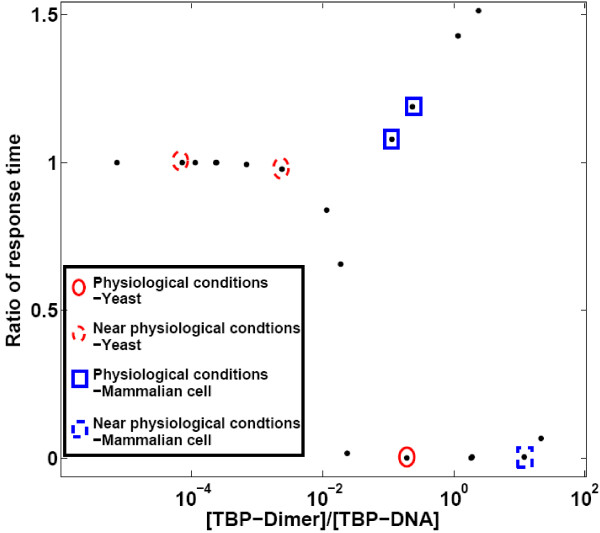
**The graph of ratio of response time in the presence of dimer and that in the absence of dimer vs ration of TBP dimer concentration to TBP-DNA complex concentration**. Unannotated points represent conditions that are not likely to be physiological (either in yeast or mammalian cells.)

It is known that TBP is (mostly) present as a dimer when not bound to DNA. We used the mathematical model to explore the role of TBP dimerization under reported physiological conditions (TBP levels, number of binding sites, dimerization and DNA binding rate constants). Equation (6), which determines the number, magnitude, and stability of steady states does not contain the dimerization (k1) and dissociation (k2) paramters. Thus, it was clear that the presence or absence of dimer does not affect the multistability of the system. Although the free and bound steady state TBP concentration was independent of the presence of dimer, the total TBP concentration was increased by amount [T_2_] ss = (k1/k2)*[T]ss^2^. The presence of dimer was found to be important in affecting the dynamics of the system. We used the model to explore in general the role of dimerization in buffering the response to transient perturbations in TBP levels for a range of total TBP and total binding site numbers. To evaluate the effect of presence of dimer on the response to perturbations in TBP levels, we compared the response time of this system (with dimer) to a hypothetical system where there is no dimerization reaction. Figure 8 shows the graph of the ratio of response time in the presence/absence of dimer as a function of the relative dimer concentration. Points near the x-axis (ratio of response time <<1) indicate conditions where the presence of dimer substantially decreases the response time.

It was observed that, (Figure 8) for typical yeast cell condition (case 9) the response time in the presence of dimer was much less than that in the absence of dimer. On the other hand for mammalian cell conditions (case 19 and 20) the response time was almost identical. For the conditions near yeast physiological conditions (case 10-12) the response time in both the 'with dimer' and 'without dimer' was almost identical. This indicated that the dimer did not always help to buffer the system against perturbations. The concentration of TBP dimer relative to TBP-DNA was found to be an important factor determining the response against perturbation. For conditions where most of TBP was present as TBP-DNA complex the response time was observed to be same. It was observed that when TBP dimer concentration was less than that of TBP-DNA, dimer dissociation did not contribute significantly to regain the free TBP steady state concentration. On the other hand, dissociation of TBP-DNA complex would help to reach the TBP steady state. In such conditions, therefore, the response time for both the systems was observed to be almost same. But two cases were observed (cases 19 and 20) where, though the TBP dimer and TBP-DNA complex concentration was similar the response time in the presence and absence of dimer were almost same. Thus, although the presence of dimer is always important for the response time when its relative concentration is high (>10) and always unimportant when its relative concentration is low (<0.01), at physiological level where the relative concentration is close to 1, it is not a good indicator of its importance to the response to perturbation in free TBP level.

## Discussion and Conclusions

We have developed a model for TBP intracellular concentration that includes autocatalytic production and dimerization. The model for TBP auto-regulation developed and used here is a continuous deterministic model that considers the positive feedback loop of TBP and non-linear self regulation through dimerization. The effect of varying the reaction rate parameters on the magnitude and the nature of the steady state solutions is explored. The effect of presence and absence of dimer is evaluated. For conditions where the number of molecules of any reactant is low, stochastic effects may play a significant role in defining the systems dynamics. This would be a logical extension to the present study.

The kinetic model of TBP revealed the possibility of a range of potential behaviours. For parameter values where the system exhibits bistability, one of the steady states is the zero-state and the other is a high-TBP state corresponding to a total TBP concentration in the 10 nanomolar range. It is seen that a physiological state resulting in low total TBP concentration (below ~0.1 nanomolar) will be in the zone of attraction of the zero-state and hence lead to cell death. Studies have shown that mRNA of many of the proteins involved in basic processes of gene expresion are maternally inherited [21,22] since the egg cell is transcriptionally silent but translationally active [23]. These maternally inherited proteins help the growth of zygote during first few cycles of cell division. The model predicts that a certain amount of TBP is needed to "jump start" its own transcription. These properties may also explain the reason for the maternal inheritance of TBP [24].

Mutants spanning important residues in the DNA binding domain and fusion with other DNA binding domains have been generated [25]. Several mutants of TBP with altered DNA binding specificity and affinity have also been reported [26]. Naturally occurring mutants of TBP associated with cancer and neurodegenerative disorders are also known [27,28]. The kinetic model of TBP presented here provides a framework for analyzing the behaviour of these mutants. For instance, it has been suggested that in case of neurodegenerative diseases the length of polyglutamine stretch is inversely correlated with binding of the TBP to DNA [28]. Bifurcation plots indicate that high-TBP state is sensitive to variation in DNA binding strength. For around 50% reduction in TBP-DNA binding strength the steady state behaviour of the system changes such that it moves from a three steady-state regime (with two stable states) into one where there is only one steady state which is the zero state. This indicates that at low values of binding rate constant the cell may not be able to survive irrespective of the initial amount of TBP present.

The model is used to test the effect of presence of dimer. It is known that TBP is present as dimer when not bound to DNA and it is believed that the dimer helps to buffer against perturbations making the system robust [19]. Our model predicts that the buffering ability depends upon the relative concentration of TBP and TBP binding sites on DNA. For some physiological conditions, therefore the dimer may not help against transient perturbation. This is an experimentally verifiable prediction, and may be of relevance in considering the effects of unmasking of TBP binding sites by chromatin organization in activation of genes.

Using the model, we have suggested explanations for experimental observations and have made testable predictions. This is an example of the utility of mathematical modelling in generating new hypotheses for complex biological systems.

## Methods

### Model development

Figure 1 shows the processes considered in the model. We lumped transcription and translation into one step, as has been assumed in other models [8,29]. Considering the non-linear nature of transcription and translation process, formation of TBP was assumed [30] to follow Hill-kinetics. Here, the use of Hill kinetics does not imply any particular mechanism but merely the fact that combining two nonlinear processes (transcription and translation) may result in a highly-nonlinear saturating dependence of protein production rate on the bound TBP concentration. We have assumed that TBP has the same affinity for all the promoter sites. The concentration of TBP-bound TBP-promoter is hence a constant fraction of the total bound TBP. Therefore, we have taken the rate of TBP synthesis as a function of the average TBP bound to all sites. TBP degradation was assumed to be first order, and all other reactions were assumed to follow mass-action kinetics. The reactions included in the model (Table 1) were reversible TBP dimerization, reversible binding of TBP to DNA, formation of TBP and TBP degradation. Jackson-Fisher and co-workers have shown that dimerization stabilizes TBP from degradation [20]. It was assumed that TBP dimers and DNA bound TBP do not undergo degradation. Based on these reactions the differential equations for the species were formulated considering the rate of formation, rate of conversion and the rate of degradation.

The mass balance equations for the species in the model are given by (1) - (5), where [T], [T_2_], [TD] and [D] represent concentration of free TBP, TBP dimer, TBP-DNA complex and unbound TBP binding sites respectively.

(1) $$\frac{d[T]}{dt}=2\times k2\times [T_{2}]-2\times k1\times {\left[T\right]}^{2}+k4\times [TD]-k3\times [T]\times [D]+\left(k0+\frac{k5\times {\left[TD\right]}^{k6}}{k7^{k6}+{\left[TD\right]}^{k6}}\right)-k8\times [T]$$

(2) $$\frac{d[T_{2}]}{dt}=k1\times {\left[T\right]}^{2}-k2\times [T_{2}]$$

(3) $$\frac{d[D]}{dt}=k4\times [TD]-k3\times [T]\times [D]$$

(4) $$\frac{d[TD]}{dt}=k3\times [T]\times [D]-k4\times [TD]$$

The concentration of total promoter sites is given as

(5) $$\left[D]+[TD]=[D_{0}\right]$$

In the absence of reaction rate constants for TBP interaction at TATA less promoters, we used the model and rate constants used by Pugh et al [17] for the TBP dimerization and DNA binding reactions. The number of TBP molecules reported for different cell types such as yeast, mammalian cell, sea urchin egg cell has wide range of 2000 to 2*10^6 ^[24,31]. Due to cell size variation, ranging from 1 μm radius for yeast to 50 μm for sea urchin egg cell leading to cell volume range of 4.17*10^-15 ^to 5.22*10^-10 ^litres, the observed physiological concentration range for TBP was found to be10^-5^M to 10^-8^M. Similarly studies showed a range for number of genes expressed in different cells, implying a minimum number for TBP binding sites in genome. For instance, there are ~3000 expressed genes in yeast cell [32] and ~10000 in mammalian cell [33]. The reported maximum number for TBP binding sites in mammalian cell is ~80,000 [34]. Hence, the physiological concentration range for TBP binding sites was found to be 10^-5^M to 10^-9^M. The details of concentration range are given in Additional file 1, Table S2A. Here we have considered a mammalian cell conditions (~25000 TBP molecules and ~25000 TBP binding sites). We have checked the effect of varying these numbers (Additional file 1, Table S3A). All the parameters used for simulation are given in Table 2. The ordinary differential equations were solved using the ode15 s stiff differential equation solver of MATLAB version 7.6.0.324 (The Mathworks, Natick, USA).

### Simulations for Dimerization effect

To study the effect of presence of dimer, a hypothetical system was considered where the dimerization reaction rate constant (k1) was set to zero. Removing the dimerization reaction would decrease the total TBP concentration by the amount equivalent to the dimer concentration. The initial condition was set to the respective high-TBP steady state in both the systems. The systems were perturbed by decreasing the free TBP concentration by 10%. Time required to regain 99% of the steady state value (i.e. to recover 90% of the perturbation) was taken as a measure of the response time of the system to perturbations. This response time was compared for the two systems to assess the effect of dimerization on the response kinetics. This study was done by varying reaction rate parameters (k5 or k7) and TBP binding sites (D_0 _= 10^-5^M to 10^-9^M) to give a range of TBP (10^-5^M to 10^-8^M) concentration. 23 different conditions were studied. Similar perturbation analysis can be done using an analytical method [35], if the system is linear near the steady state. For this system we carried out such analysis and found that the results are in agreement with the computational study (Additional file 1, Figure S1A).

### Steady state solution

At steady state, the concentration of each species remains constant. Setting the left hand side of equations 1-4 to zero and simplifying resulted in a nonlinear function (f) of one variable, [T],

(6) $$f([T])=(k8\times [T]-k0)\times \left(k7^{k6}+{\left(\frac{\left[T]\times [D_{0}\right]}{\frac{k4}{k3}+[T]}\right)}^{k6}\right)-k5\times {\left(\frac{\left[T]\times [D_{0}\right]}{\frac{k4}{k3}+[T]}\right)}^{k6}=0$$

In the absence of basal expression i.e. when k0 = 0 Ms^-1^, the equation became,

(7) $$f([T])=\left(k8\times [T]\right)\times \left(k7^{k6}+{\left(\frac{\left[T]\times [D_{0}\right]}{\frac{k4}{k3}+[T]}\right)}^{k6}\right)-k5\times {\left(\frac{\left[T]\times [D_{0}\right]}{\frac{k4}{k3}+[T]}\right)}^{k6}=0$$

The system of 4 equations (equations 1 to 4) was reduced to 3 equations and non-dimensionalized using [D_0_] as reference concentration and 1/k8 as reference time (equations 1.4 to 1.6). The non-dimensional parameters are represented in capital letter (e.g. K1 is non dimensional parameter obtained from k1). The details are given in Additional file 1. Analytical expressions for steady state levels of TBP were obtained. For the condition where k0 = 0 Ms^-1^, the expression for critical k3 and critical k5, below which there existed only one steady state, was obtained. The analysis was done in Mathematica version 7.0.1.0 (Wolfram Research, Champaign, USA).

## Competing interests

The authors declare that they have no competing interests.

## Authors' contributions

CJG and BP initiated the study; SAG and VK carried out the steady state simulation; SAG carried out the algebraic analysis, transient studies, formulated the dimerization effects model and carried out parameter variation simulations; SAG, RR, BP and CJG analyzed the data and related simulations to experimental observations; SAG wrote the initial draft of the manuscript; SAG, RR, BP and CJG participated in writing the final version of the manuscript. All authors read and approved the final manuscript.

## Reviewers' comments

### Reviewer's report 1

#### Tomasz Lipniacki, Institute of Fundamental Technological Research, Poland

The Authors proposed a simple model of TATA binding protein (TBP) auto regulation. The model is based on the assumption that expression of TBP gene requires binding of TBP protein to TATA box of TBP gene. The Authors justified this key assumption by the observation that TBP is required by all the three types of RNA eukaryotic polymerases (Ref [13], Hernandez 1993). The assumed TBP positive autoregulation leads to the positive feedback, which results, for a broad range of parameters, in bistability in TBP levels. This is the main finding of the manuscript. The system has two stable steady states zero - corresponding to non-viable cells and the positive corresponding to viable cells.

The Authors, following to some extent Ref. [14], discussed also the role of TBP dimerisation, which, since dimers may not bind DNA, introduce the negative feedback and stabilizes the level of TBP-DNA complexes.

Major comments

Major Comment 1:

In my opinion the key assumption that TBP protein is necessary for TBP expression is not sufficiently justified - the Authors should put more effort in justifying the assumption, while they only refer to 1993 review. The bistability following from this assumption is rather unexpected for the protein which is crucial for cell survival.

Authors' response:

We have included more references (Cormack B.P and Struhl K.1992, and White R.J et al 1992) to justify the requirement of TBP by all the three RNA polymerases and for different types of promoters. Also, following the analysis, we find that a very small basal transcription rate also leads to similar results hence the absolute requirement (k0 = 0) of TBP for bistability is no longer essential.

Major Comment 2:

The Eq. 1 seems to be incorrect: why the synthesis rate of TBP is given by the concentration of TPB-DNA complexes (variable TD). It should depend on the TBP gene state alone - not on the concentration of TBP bound to all the other gene promoters.

Authors' response:

We have assumed that there is no preferential binding of TBP to its own promoter site over other promoter sites, and therefore have taken the rate as a function of the average TBP bound to all sites. For particular case of TBP gene, the complex TD' will be a linear function of TD, i.e the fraction [TD'] = [TD]*1/[D0]. Thus the rate of synthesis of TBP would be a function of TD' resulting in a same functional form as of eq.1. We have modified the text to better communicate this assumption.

Minor comments

Minor comment1:

In Eq. 2 there is no degradation term - this could be the reasonable assumption if TBP dimers are particularly stable, but should be discussed. Similarly, it follows from Eq. 4 that TBP degradation in TBP-DNA complexes is neglected.

Authors' response:

We have included the statement stating these assumptions and a reference (Jackson-Fisher et al, 1999) in support of the assumption.

Minor comment2:

Discussing Figure 2, the Authors wrote that in the presence of basal TBP transcription rate k0>0 the system is monostable. If the system is bistable for k0 = 0, then from the continuity it follows that there exists sufficiently small k_c, such that for k0 < k_c the system is bistable. I agree that this critical k_c may be very small - which makes the solid justification of assumption that k0 = 0 crucial.

Authors' response:

Following several editors' comments, we have carried out an analysis of the steady state solutions in addition to the previous perturbation analysis and derived an expression for the critical value of k0 such that below this value (k0_c) the system is bistable. The value of k0_c is observed to be 0.008*k5 (4*10^-15^Ms^-1^). Justification for assumption of k0 = 0 is discussed in response to major comment 1.

Minor comment3:

In Figure 3 the Authors present log-log plots in which concentrations vary by more than 20 orders of magnitude this is biologically unrealistic and misleading.

Authors' response:

The previous plots show values of ~10^-20 ^for species concentration, which essentially indicate the zero TBP steady state. We have modified the plot as suggested and added text to clarify that the lower steady state that cannot be shown on a log-log plot is the zero state.

### Reviewer's report 2

#### James Faeder, Department of Computational and Systems Biology, University of Pittsburgh School of Medicine

The paper presents a simple ordinary differential equation model for the expression of TATA Box protein (TBP). Promoter activity is assumed to depend on the binding of TBP in the monomeric form, which is countered by the formation of TBP dimers that do not bind the promoter. The analysis shows that depending on the parameters that govern the strength of the positive feedback, either one or two steady states may be stable. The steady state with non-zero TBP expression disappears in some regions of the parameter space, which is interpreted as evidence that mutations that reduce the strength of TBP-promoter interactions or activity can lead to non-viability. The effect of dimer formation on the kinetics of responses to changes in TBP concentration is also studied. It is also found that the level of TBP in dimeric form relative to the amount bound to promoter has a strong, though not necessarily decisive, influence on the response time. A significant effort is made to estimate parameter values relevant for a wide range of cell types. The analysis is interesting and the model, despite some potential flaws noted below, represents a significant contribution that may serve as the foundation for future work.

Comment 1

I found the title of the paper, "A kinetic model of TBP auto-regulation suggests bistable nature of TBP expression," to be confusing because it seems to imply that TBP expression may be bimodal. This confusion was increased by the abstract because the existence of a second stable steady state is described only as "corresponding to unviable cells." Until I read the Results, I thought the unviable steady state was the result of too high an expression level of TBP rather than zero expression of TBP. At any rate, I would suggest that a clearer title would be "A kinetic model of TBP auto-regulation exhibits bistability" or simply, ""A kinetic model of TBP auto-regulation", because the bistability is only one finding of the study and leaves out the role of dimerization.

Authors' response:

We agree that "A kinetic model of TBP auto-regulation exhibits bistability" is a better choice for the title and have changed the previous title "A kinetic model of TBP auto-regulation suggests bistable nature of TBP expression".

Comment 2

The modeling of TBP production using a Hill function of the bound promoter concentration ([TD]) is unconventional and should be justified. The "nonlinear nature of the transcription and translation process" (first paragraph of Methods - Model development) is not sufficient explanation for assuming that the rate of protein expression is a highly nonlinear function of the amount of transcription factor that is bound. Normally, promoter activity is taken to be proportional to the fractional occupancy of a given promoter state, with the nonlinearity arising from multivalency and cooperativity of transcription factor binding (see, e.g., U. Alon, An Introduction to Systems Biology, Appendix B). This is potentially a serious deficiency of the model, because, as is shown in Figure 6, when the Hill coefficient (k6) is one or less, the high-TBP can replace the zero-TBP state as the only stable steady state. This would be expected to dramatically alter, for example, the region of viability (where non-zero TBP expression is stable) in the parameter space plotted in Figure 7. This assumption also disturbs the relationship between the TBP-DNA affinity constants, which are taken from experimental data, and the final expression levels of TBP. The former are assumed to correspond to single-site affinity constants, whereas the later are essentially assumed to be the consequence of multivalent interactions involving TBP, DNA, and possibly additional proteins that form the activation complex.

Authors' response:

We have assumed that the rate of TBP synthesis is a function of the average TBP bound to promoter sites. The TBP bound promoter represents promoter activity. To represent the non-linear nature of transcription and translation processes involved in TBP synthesis, Hill function is assumed, as has been done by other researchers (Becksei et al, EMBO J, 2001). The function represents the phenomenological model not the mechanistic details of the process. Thus beyond the assumption that the relationship between protein production rate and TBP's association with its promoter binding is similar to a plot that is obtained from a Hill-kinetics-function, there is no intent to claim that any specific mechanism is responsible for the nature of the function, or that any of the Hill parameters are related to the actual mechanism (for instance k6 to the extent of multimerization etc). We acknowledge that this implies that the parameters are therefore assumptions and have carried out a numeric and now analytic study showing the effect of these parameters on the qualitative and quantitative results. We have also clarified this assumption by modifying the text to include an explicit statement that there is no mechanism implied by the assumption of Hill kinetics, and including a reference to a previous instance where a similar function has been used.

Comment 3

The analysis of the role of dimerization in determining the response times to perturbation of the TBP level seems incomplete. The relative concentrations of TBP in dimers and TBP bound to DNA does not seem to be the decisive parameter, as shown clearly in Figure 8, where at the same ratios dramatic differences in the role of the dimer mechanism are observed. What other differences in the parameters between mammalian and yeast cells could account for this observation? In addition, as I note under minor issues, there seem to be some discrepancies between the figure and the description provided in the Results section. There should also be some mention of the effect that removing the dimerization reaction has on the overall levels of TBP expression. How do the negative effects of dimerization interact with the positive feedback due to autoregulation?

Authors' response:

The relative concentrations of TBP in dimers and TBP bound to DNA can be a decisive parameter in a certain range of relative concentrations as mentioned. The other parameters which contribute the distribution of TBP in dimers and as TBP bound to DNA are rate constants in TBP synthesis reaction, k5 and k7. This results in the differences in the response time in the presence and absences of TBP dimer.

As per the suggestion we have added the statement mentioning that removing the dimerization reaction would decrease the total steady state TBP concentration by the amount equivalent to the dimer concentration, but not affect the steady state free monomeric and bound TBP levels, or the existence or nature of the multiple steady states

Comment 4

Why aren't stochastic effects considered? It seems quite likely that TBP expression levels would undergo large fluctuations if the copy number of TBP genes is low, and it is also possible that stochastic effects could restore the stability of the high-TBP state under some conditions.

Authors' response:

We agree that such a study would be of considerable importance and interest (and we are currently carrying out the simulations as part of a larger study on the effect of stochasticity) but we feel that the current results are by themselves also of interest. We have included a statement in the discussion pointing to the need for stochastic analysis.

### Reviewer's report 3

#### Anna Marciniak-Czochra, University of Heidelberg,Institute of Applied Mathematics, Germany

The paper is devoted to mathematical modeling of the effects of TATA Binding Protein (TBP) dimerization on the TBP expression levels. The model is given in the form of 4 ordinary differential equations describing time dynamics of TBP monomers, dimers, bound TBP-DNA complexes and free binding sites. The authors show that under some conditions on parameters, the model exhibits multistability of the steady states, which lead to the switches in the model dynamics depending on the perturbation of the parameters. The model is interesting and shows the effects of the existence of multiple steady states on the system dynamics. Model predictions are compared to the experimental observations and the biologically relevant conclusions are drawn. For example, the model suggests that a certain minimal amount of TBP is needed to start its production. It allows also to test the hypothesis on the role of dimerization in the stabilization of the process. Unfortunately in the current version I cannot recommend this manuscript for publication. The manuscript needs some revisions, mostly related to the structure of the presentation.

Comment 1:

My major criticism concerns the analysis of the model. The system of 4 ODEs can be reduced to 3 ODEs using a conservation law for the concentration of free and bound binding sites, and then nondimensionalized. Analysis of the different cases should be done more systematically. Conditions for the existence of 3 steady states should be found. Presentation of the linear stability analysis on pages 19-20 should be improved. It is not necessary to repeat well known facts, such as the general form of the solution (which in fact is true only under some conditions), and calculated eigenvectors. Formula (1.8 Now, numbered as 1.13) is completely meaningless. I my opinion linear stability analysis should be presented if it leads to any conclusions about stability of the steady states. Otherwise, only numerical analysis should be presented.

Authors' response:

We have tried to imagine as our target audience experimental biologists who might be interested in the results using the parameters we believe are representative of physiological conditions, and hence had not included the mathematical analysis. However we agree that such an analysis would add value. As suggested, the system of 4 ODEs was reduced to 3 ODEs and nondimensionalized. The conditions for existence of three steady states were found out and added in the results.

We feel that although the nature of the system (3 equations, mostly linear or mass-action kinetics) would imply that most results would be elementary for people with a mathematics training. However they may not be as obvious to other biologists who may not have a formal training in mathematics, and feel that elaboration of the steps carried out for the perturbation analysis may be of interest to such readers. Hence we have opted to retain the details (in Additional file 1).

Comment 2:

It should be better explained which effects come from the dimerization process and which are related to the different assumptions, such as the Hill-type nonlinearity in the term describing TBP transcription. In particular, if the multistability results from the Hill-type kinetics, its biological relevance should be justifed.

Authors' response:

We added the statements discussing the effect of dimer on the steady states and the dynamics of the system. The presence of dimer in some cases was found to be important for making the system robust against perturbations. It affects the kinetics of how the system reaches the steady state as evident from the response time analysis. But the presence or absence of dimer does not affect the multistability of the system as observed from equation (6).

To represent the non-linear nature of transcription and translation processes involved in TBP synthesis, Hill function is assumed which represents the phenomenological model as discussed earlier. We have changed the Hill co-operativity coefficient and observed the effect on multistability (Figure 7).

Minor remarks:

Comment 1:

Figure 1 is not informative enough. The fonts are too small and the picture is clear only if one knows what it presents. The fgure should be improved.

Authors' response:

We modified the figure accordingly.

Comment 2:

The estimates for the concentration of TBP binding sites, etc (on page 5) should be better explained. The given explanation is not clear.

Authors' response:

We have modified the statement explaining the concentration ranges. Details of calculation for concentrations are given in Additional file 1.

Comment 3:

The quality of all figures is not satisfactory, e.g., fonts are too small.

Authors' response:

We modified the figures accordingly.

## Supplementary Material

Additional file 1 **Details of perturbation studies for TBP system**. Click here for file
