# Supplementary material for: A kinetic model of TBP auto-regulation exhibits bistability
Source: Biol Direct. 2010 Aug 5;5:50. doi: 10.1186/1745-6150-5-50 (PMC2928763; doi:10.1186/1745-6150-5-50)
Supplement: Additional file 1 — Details of perturbation studies for TBP system. [file 1745-6150-5-50-S1.PDF]

**Analysis of conditions for existence of multiple steady states**

The TBP system can be reduced to a system with three variables, TBP, TBP dimer and TBP-DNA complex using the conservation relation for total TBP binding sites (equation 5).

The equations for the three variable system are,

$$f1=\frac{d[T]}{dt}=-2\times k1\times[T]^2+2\times k2\times[T_2]-k3\times[T]\times([D_0]-[TD])+k4\times[TD]+\frac{k5\times[TD]^{k6}}{k7^{k6}+[TD]^{k6}}-k8\times[T] \quad (1.1)$$

$$f2=\frac{d[T_2]}{dt}=k1\times[T]^2-k2\times[T_2] \quad (1.2)$$

$$f3=\frac{d[TD]}{dt}=k3\times[T]\times([D_0]-[TD])-k4\times[TD] \quad (1.3)$$

The above system of three ODE (1.1-1.3) was nondimensionalized. $[D_0]$ is used as reference concentration and 1/k8 is used as reference time. The equations are as follows.

$$\frac{d[T]}{dt}=-2\times K1\times[T]^2+2\times K2\times[T_2]-K3\times[T]\times(1-[TD])+K4\times[TD]+\frac{K5\times[TD]^{k6}}{K7^{k6}+[TD]^{k6}}-[T] \quad (1.4)$$

$$\frac{d[T_2]}{dt}=K1\times[T]^2-K2\times[T_2] \quad (1.5)$$

$$\frac{d[TD]}{dt}=K3\times[T]\times(1-[TD])-K4\times[TD] \quad (1.6)$$

Where, the concentrations and parameters (K1 to K7) are dimensionless (Table 1A).

Conditions for existence of multiple steady states were found out by analyzing the expression for steady state level of TBP, using the above dimensionless equations. Following four conditions were considered for different K0 and k6 values.

1. K0 ≠0, K0 > K0_c, k6 = 2
   Three steady states were observed. The expression for steady state level of TBP is available as Mathematica file on request.

2. K0 = 0, k6 = 2
   Three steady states were observed.
   $T_{ss1}=0$
   $$T_{ss2}=\frac{K5-2KK7^2-\sqrt{K5^2-4K^2K7^2-4KK5K7^2}}{2(1+K7^2)}$$
   $$T_{ss3}=\frac{K5-2KK7^2+\sqrt{K5^2-4K^2K7^2-4KK5K7^2}}{2(1+K7^2)}$$

   Where, K = K4/K3

From the above expressions, it was observed that multiple real non-negative steady states would exist when $K5 > 2KK7^2$.
On simplification, the expression for $T_{ss2}$ leads to

$$T_{ss2} = \frac{K5 - 2KK7^2 - \sqrt{(K5 - 2KK7^2)^2 - 4K^2K7^2(1 - K7^2)}}{2(1 + K7^2)}$$

It was observed from the expression that K7 < 1, would result in a non-negative steady state. Therefore for the above conditions 3 non-negative real steady states were observed.

From the bifurcation diagrams it was observed that above a particular value of k3 or k5 the system is multistable. At the critical value of k3 or k5, the two steady states $T_{ss2}$ and $T_{ss3}$ were observed to be equal. Thus the expression for critical values of k3 (K3_c) and k5 (K5_c) was obtained by equating the expression for $T_{ss2}$ and $T_{ss3}$.

$$T_{ss2} = T_{ss3}$$

Which gives,

$$\frac{-\sqrt{K5^2 - 4K^2K7^2 - 4KK5K7^2}}{1 + K7^2} = 0 \qquad (1.7)$$

Equivalent to,

$$K5^2 - 4K^2K7^2 - 4KK5K7^2 = 0 \qquad (1.8)$$

From the above equation K3_c was obtained,

$$K3\_c = \frac{2\left(4K5K7^2 + \sqrt{K4^2K5^2K7^2 + K4^2K5^2K7^4}\right)}{K5^2}$$

From the same equation (1.13), K5_c was obtained,

$$K5\_c = 2\left(KK7^2 + \sqrt{K^2K7^2 + K^2K7^4}\right)$$

3. $K0 \neq 0$, k6 = 1
   Two steady states were observed.

$$T_{ss1} = \frac{K0 + K5 - KK7 + K0K7 - \sqrt{4KK0K7(1 + K7) + (K0 + K5 - KK7 + K0K7)^2}}{2(1 + K7)}$$

$$T_{ss2} = \frac{K0 + K5 - KK7 + K0K7 + \sqrt{4KK0K7(1 + K7) + (K0 + K5 - KK7 + K0K7)^2}}{2(1 + K7)}$$

At K0 << K and K5 > KK7, (K0+K5-KK7+K0K7), results in a positive term.
On simplification, the expression for $T_{ss1}$ leads to

$$T_{ss1} = \frac{(K0 + K5 - KK7 + K0K7) - \sqrt{(K0 + K5 - KK7 + K0K7)^2 + 4K0(1 + K7)KK7}}{2(1 + K7)}$$

This indicated that $T_{ss1}$ would be the negative steady state and $T_{ss2}$ would be non-negative steady state.

4. K0 = 0, k6 = 1
   Two steady states were observed.

$$T_{ss1} = 0$$
$$T_{ss2} = \frac{K5 - KK7}{1 + K7}$$

   K5 > KK7, would result in two real steady states.

**Perturbation Study for TBP system**

In the neighbourhood of steady state, for the above system (equations 1.1 to 1.3), the rate of change of perturbation ($\sigma_i$) for variable i, can be given as [1],

$$\frac{d\sigma_T}{dt} = \frac{\partial f1}{\partial [T]}\sigma_T + \frac{\partial f1}{\partial [T_2]}\sigma_{T_2} + \frac{\partial f1}{\partial [TD]}\sigma_{TD} = a_{11} \times \sigma_T + a_{12} \times \sigma_{T_2} + a_{13} \times \sigma_{TD} \quad (1.9)$$

$$\frac{d\sigma_{T_2}}{dt} = \frac{\partial f2}{\partial [T]}\sigma_T + \frac{\partial f2}{\partial [T_2]}\sigma_{T_2} + \frac{\partial f2}{\partial [TD]}\sigma_{TD} = a_{21} \times \sigma_T + a_{22} \times \sigma_{T_2} + a_{23} \times \sigma_{TD} \quad (1.10)$$

$$\frac{d\sigma_{TD}}{dt} = \frac{\partial f3}{\partial [T]}\sigma_T + \frac{\partial f3}{\partial [T_2]}\sigma_{T_2} + \frac{\partial f3}{\partial [TD]}\sigma_{TD} = a_{31} \times \sigma_T + a_{32} \times \sigma_{T_2} + a_{33} \times \sigma_{TD} \quad (1.11)$$

Where, $a_{ij}$ are elements of Jacobian matrix J,

$$\begin{pmatrix} -4 \times k1 \times [T] - k3 \times [D_0] + k3 \times [TD] - k8 & 2 \times k2 & k3 \times [T] + k4 + \dfrac{k5 \times k6 \times k7^{k6} \times [TD]^{k6-1}}{\left(k7^{k6} + [TD]^{k6}\right)^2} \\ 2 \times k1 \times [T] & -k2 & 0 \\ k3 \times [D_0] - k3 \times [TD] & 0 & -k3 \times [T] - k4 \end{pmatrix}$$

The general solution for the above system is given by,

$$\sigma(t) = c1 \times e^{\lambda_1 t} \times v_1 + c2 \times e^{\lambda_2 t} \times v_2 + c3 \times e^{\lambda_3 t} \times v_3 \quad (1.12)$$

Where $\lambda_1$, $\lambda_2$ and $\lambda_3$ are three eigenvalues and $v_1$, $v_2$ and $v_3$ are corresponding eigenvectors of J. Analytical expression for eigenvectors was obtained in terms of eigenvalues and reaction rate constants.

The eigenvectors are,

$$v_1 = \begin{pmatrix} \dfrac{a_{12} + a_{13}\left(\dfrac{(\lambda_1 - a_{11})(\lambda_1 - a_{22}) - a_{12} \times a_{21}}{a_{13} \times a_{21} + a_{23}(\lambda_1 - a_{11})}\right)}{(\lambda_1 - a_{11})} \\ 1 \\ \dfrac{(\lambda_1 - a_{11})(\lambda_1 - a_{22}) - a_{12} \times a_{21}}{a_{13} \times a_{21} + a_{23}(\lambda_1 - a_{11})} \end{pmatrix}, \quad v_2 = \begin{pmatrix} \dfrac{a_{12} + a_{13}\left(\dfrac{(\lambda_2 - a_{11})(\lambda_2 - a_{22}) - a_{12} \times a_{21}}{a_{13} \times a_{21} + a_{23}(\lambda_2 - a_{11})}\right)}{(\lambda_2 - a_{11})} \\ 1 \\ \dfrac{(\lambda_2 - a_{11})(\lambda_2 - a_{22}) - a_{12} \times a_{21}}{a_{13} \times a_{21} + a_{23}(\lambda_2 - a_{11})} \end{pmatrix},$$

$$v_3 = \begin{pmatrix} \dfrac{a_{12} + a_{13}\left(\dfrac{(\lambda_3 - a_{11})(\lambda_3 - a_{22}) - a_{12} \times a_{21}}{a_{13} \times a_{21} + a_{23}(\lambda_3 - a_{11})}\right)}{(\lambda_3 - a_{11})} \\ 1 \\ \left(\dfrac{(\lambda_3 - a_{11})(\lambda_3 - a_{22}) - a_{12} \times a_{21}}{a_{13} \times a_{21} + a_{23}(\lambda_3 - a_{11})}\right) \end{pmatrix}$$

In the perturbation study, the initial perturbation for TBP is 10% of steady state free TBP concentration and zero for the other two species. From these initial conditions, analytical expressions for c1, c2 and c3 were obtained.

From, equation 1.12, and the above expression,

$$\sigma_T(t) = B_{11} \times e^{\lambda_1 t} + B_{12} \times e^{\lambda_2 t} + B_{13} \times e^{\lambda_3 t} \tag{1.13}$$

Where, $B_{11} = \dfrac{-(x(k2+\lambda_1)(k2+[D_0]\times k3+k8+4\times k1\times[T]-k3\times[TD]+\lambda_2+\lambda_3))}{(\lambda_1-\lambda_2)(\lambda_1-\lambda_3)}$,

$B_{12} = \dfrac{x(k2+\lambda_2)(k2+[D_0]\times k3+k8+4\times k1\times[T]-k3\times[TD]+\lambda_1+\lambda_3)}{(\lambda_1-\lambda_2)(\lambda_2-\lambda_3)}$ and

$B_{13} = \dfrac{-(x(k2+\lambda_3)(k2+[D_0]\times k3+k8+4\times k1\times[T]-k3\times[TD]+\lambda_1+\lambda_2))}{(\lambda_1-\lambda_2)(\lambda_2-\lambda_3)}$

Here, x is initial perturbation in [T].

In this case the response time is the time required to recover 90% of the perturbation. In this case, therefore, $\sigma_T(t)$ is 10% of the initial perturbation.

We compared the response time obtained for the two systems. The response time obtained with this method found to be almost same to that obtained by computational method (figure 1A). The correlation coefficient between the two methods was 0.99.

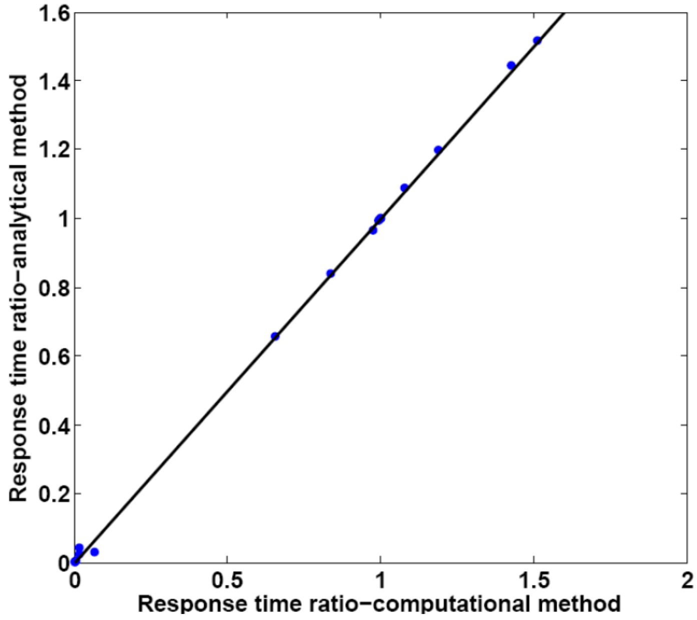


Figure 1A: Graph of ratio of response time by analytical method vs ratio of response time by computational method.

Table 1A: Dimensionless parameter

| **Parameter with dimension** | **Dimensionless parameter** |
|---|---|
| $k1(M^{-1}s^{-1})$ | $K1 = k1\frac{D_0}{k8}$ |
| $k2(s^{-1})$ | $K2 = \frac{k2}{k8}$ |
| $k3(M^{-1}s^{-1})$ | $K3 = k3\frac{D_0}{k8}$ |
| $k4(s^{-1})$ | $K4 = \frac{k4}{k8}$ |
| $k5(Ms^{-1})$ | $K5 = \frac{k5}{D_0 k8}$ |
| $k7(M)$ | $K7 = \frac{k7}{D_0}$ |
| $k0(Ms^{-1})$ | $K0 = \frac{k0}{D_0 k8}$ |

Table 2A: Concentration range for total TBP and TBP binding sites

| | **Yeast Cell**<br>Radius: 1 µm [2] and references therein<br>Volume: $4.17*10^{-15}$ lit | **Mammalian Cell**<br>Radius: 7.5 µm [3] (http://bionumbers.hms.harvard.edu)<br>Volume: $1.76*10^{-12}$ lit | **Sea urchin egg Cell**<br>Radius: 50 µm [4]<br>Volume: $5.22*10^{-10}$ lit |
|---|---|---|---|
| TBP Concentration | 4800 molecules = $1.91*10^{-6}$M<br>20,000 molecules = $7.96*10^{-6}$M<br>$2*10^{9}$ molecules = 0.7M | 4800 molecules = $4.5*10^{-9}$M<br>20,000 molecules = $1.88*10^{-8}$M<br>$2*10^{9}$ molecules = $1.9*10^{-3}$M | 4800 molecules = $1.5*10^{-11}$M<br>20,000 molecules = $6.3*10^{-11}$M<br>$2*10^{9}$ molecules = $6.3*10^{-6}$M |
| TBP binding site concentration | 3000 sites=$1.19*10^{-6}$M<br>10000 sites=$3.98*10^{-6}$M<br>80000 sites =$3.185*10^{-5}$M | 3000 sites=$2.8*10^{-9}$M<br>10000 sites=$9.4*10^{-9}$M<br>80000 sites =$7.5*10^{-8}$M | 3000 sites=$9.5*10^{-12}$M<br>10000 sites=$3.2*10^{-11}$M<br>80000 sites =$2.5*10^{-10}$M |

Table 3A: Table states range of $D_0$ concentration, k5, k7 and corresponding total TBP concentration, sensitivity to single parameter change, response time ratio and the ratio of dimer concentration to TBP-DNA complex concentration.

| Sr. No. | D0 (M) | k5 (M/s) | k7 (M) | Total TBP (M) | Decrease in high-TBP state concentration | | Ratio of response time by computational method | $[T_2]/[TD]$ |
|---|---|---|---|---|---|---|---|---|
| | | | | | Sensitivity to k3 | Sensitivity to k6 | | |
| 1 | 2.50E-05 | 5.00E-11 | 1.25E-08 | 0.000117 | 0.08% | 0.08% | 2.62E-04 | 1.83E+00 |
| 2 | 2.50E-05 | 5.00E-12 | 1.25E-08 | 2.53E-05 | 2.69% | not detectable | 6.57E-01 | 1.88E-02 |
| 3 | 2.50E-05 | 5.00E-13 | 1.25E-05 | 1.72E-05 | 43.00% | 6.53% | 9.99E-01 | 1.14E-04 |
| 4 | 2.50E-05 | 5.00E-13 | 1.25E-06 | 1.93E-05 | 18.00% | 1.45% | 9.98E-01 | 2.35E-04 |
| 5 | 2.50E-05 | 5.00E-13 | 1.25E-07 | 1.93E-05 | 18.00% | 0.10% | 9.98E-01 | 2.37E-04 |
| 6 | 2.50E-05 | 5.00E-13 | 1.25E-08 | 1.93E-05 | 18.00% | 0.05% | 9.98E-01 | 2.37E-04 |
| 7 | 2.50E-05 | 5.00E-13 | 1.25E-09 | 1.93E-05 | 19.00% | not detectable | 9.98E-01 | 2.37E-04 |
| 8 | 2.50E-05 | 5.00E-14 | 1.25E-08 | 6.31E-06 | 42.00% | 0.14% | 1.00E+00 | 7.23E-06 |
| 9 | 2.50E-06 | 5.00E-12 | 1.25E-08 | 3.41E-06 | 1.99% | 0.29% | 2.14E-03 | 1.88E-01 |
| 10 | 2.50E-06 | 5.00E-13 | 1.25E-07 | 1.94E-06 | 18.00% | 1.03% | 9.76E-01 | 2.35E-03 |
| 11 | 2.50E-06 | 5.00E-13 | 1.25E-08 | 1.94E-06 | 18.00% | 0.15% | 9.76E-01 | 2.37E-03 |
| 12 | 2.50E-06 | 5.00E-14 | 1.25E-08 | 6.32E-07 | 42.00% | 1.45% | 9.99E-01 | 7.23E-05 |
| 13 | 2.50E-07 | 5.00E-12 | 1.25E-08 | 1.22E-06 | 0.50% | 7.06% | 2.98E-03 | 1.87E+00 |
| 14 | 2.50E-07 | 5.00E-13 | 1.25E-07 | 1.81E-07 | 43.93% | 5.20% | 8.37E-01 | 1.14E-02 |
| 15 | 2.50E-07 | 5.00E-13 | 1.25E-08 | 2.09E-07 | 17.41% | 1.96% | 1.58E-02 | 2.35E-02 |
| 16 | 2.50E-07 | 5.00E-13 | 1.25E-09 | 2.09E-07 | 17.24% | 0.23% | 1.65E-02 | 2.37E-02 |
| 17 | 2.50E-07 | 5.00E-14 | 1.25E-08 | 6.20E-08 | 47.40% | 12.11% | 9.93E-01 | 6.87E-04 |
| 18 | 2.50E-08 | 5.00E-12 | 1.25E-08 | 6.44E-07 | 3.00% | 28.00% | 4.07E-03 | 1.18E+01 |
| 19 | 2.50E-08 | 5.00E-13 | 1.25E-08 | 2.56E-08 | 47.44% | 9.15% | 1.08E+00 | 1.14E-01 |
| 20 | 2.50E-08 | 5.00E-13 | 1.25E-09 | 3.51E-08 | 10.41% | 4.76% | 1.19E+00 | 2.35E-01 |
| 21 | 2.50E-09 | 5.00E-11 | 1.25E-08 | 1.21E-07 | 33.70% | 21 times increase | 6.57E-02 | 2.11E+01 |
| 22 | 2.50E-09 | 5.00E-13 | 1.25E-09 | 1.01E-08 | 52.02% | 22.70% | 1.43E+00 | 1.14E+00 |
| 23 | 2.50E-09 | 5.00E-13 | 1.25E-10 | 1.77E-08 | 2.25% | 10.15% | 1.51E+00 | 2.35E+00 |

## References


1. Heinrich R, Rapoport SM, Rapoport TA: **Metabolic regulation and mathematical models.** *Prog Biophys Mol Biol* 1977, **32:**1–82.
2. Misirli Z, Oner ET, Kirdar B: **Real imaging and size values of Saccharomyces cerevisiae cells with comparable contrast tuning to two environmental scanning electron microscopy modes.** *Scanning* 2007, **29:**11-19.
3. Ron Milo PJaMS: **BioNumbers.** May 2007.
4. Alberts B, Bray D, Lewis J, Raff M, Roberts K, Watson JD: *Molecular biology ofthe cell.* 2002.
